# Prevalence of Antibiotic and Heavy Metal Resistance Determinants and Virulence-Related Genetic Elements in Plasmids of *Staphylococcus aureus*

**DOI:** 10.3389/fmicb.2019.00805

**Published:** 2019-04-24

**Authors:** Michal Bukowski, Rafal Piwowarczyk, Anna Madry, Rafal Zagorski-Przybylo, Marcin Hydzik, Benedykt Wladyka

**Affiliations:** Department of Analytical Biochemistry, Biophysics and Biotechnology, Jagiellonian University, Kraków, Poland

**Keywords:** antibiotic resistance (AMR), *Staphylococcus aureus*, plasmid, heavy metal resistance, virulence factor, toxin–antitoxin (TA)

## Abstract

The use of antibiotics on a mass scale, particularly in farming, and their release into the environment has led to a rapid emergence of resistant bacteria. Once emerged, resistance determinants are spread by horizontal gene transfer among strains of the same as well as disparate bacterial species. Their accumulation in free-living as well as livestock and community-associated strains results in the widespread multiple-drug resistance among clinically relevant species posing an increasingly pressing problem in healthcare. One of these clinically relevant species is *Staphylococcus aureus*, a common cause of hospital and community outbreaks. Among the rich diversity of mobile genetic elements regularly occurring in *S. aureus* such as phages, pathogenicity islands, and staphylococcal cassette chromosomes, plasmids are the major mean for dissemination of resistance determinants and virulence factors. Unfortunately, a vast number of whole-genome sequencing projects does not aim for complete sequence determination, which results in a disproportionately low number of known complete plasmid sequences. To address this problem we determined complete plasmid sequences derived from 18 poultry *S. aureus* strains and analyzed the prevalence of antibiotic and heavy metal resistance determinants, genes of virulence factors, as well as genetic elements relevant for their maintenance. Some of the plasmids have been reported before and are being found in clinical isolates of strains typical for humans or human ones of livestock origin. This shows that livestock-associated staphylococci are a significant reservoir of resistance determinants and virulence factors. Nevertheless, nearly half of the plasmids were unknown to date. In this group we found a potentially mobilizable plasmid pPA3 being a unique example of accumulation of resistance determinants and virulence factors likely stabilized by a presence of a toxin–antitoxin system.

## Introduction

Growing antibiotic resistance among clinically relevant bacteria is currently becoming a grave concern of global health. The shrinking array of effective antibacterial drugs elevates mortality and increases frequency and scale of outbreaks (De Jager et al., 2015; Strauß et al., 2017; Dogramachy, 2018; Gu et al., 2018; Douglas et al., 2019). One of the most commonly addressed bacterial species in this context is *Staphylococcus aureus*. As an opportunistic pathogen *S. aureus* can colonize healthy individuals and reside asymptomatically as a commensal. The estimated carriage rate in communities reaches between 25 and 30% (Charlebois et al., 2002; Levy et al., 2005; Kuehnert et al., 2006; Li et al., 2014; Morgenstern et al., 2016). Regardless of its commensal nature, in predisposed individuals, such as newborns, young children, the elderly, immuno-compromised, post-surgical, or hospitalized ones, *S. aureus* is often a cause of difficult to treat and not rarely fatal as well as chronic infections, especially in clinical setting (Anstead et al., 2014; Savini et al., 2016). Community and livestock-associated *S. aureus* populations are of utmost importance for genetic elements exchange, in particular for dissemination of antibiotic determinants, and thus reservoirs of potentially life-threatening strains (Vandenesch et al., 2003; Armand-Lefevre et al., 2005; Voss et al., 2005; Nimmo, 2012; Planet et al., 2013; Strauß et al., 2017). For all these reasons *S. aureus* is listed by the World Health Organization as one among several bacterial species of high clinical relevance. These species form a group called ESKAPE (*Enterococcus faecium*, *S. aureus*, *Klebsiella pneumoniae*, *Acinetobacter baumannii*, *Pseudomonas aeruginosa*, and *Enterobacter*). Each of them poses a serious threat to people’s health (Pendleton et al., 2013). With regard to *S. aureus*, the greatest concerns are methicillin-resistant *S. aureus* (MRSA), resistant to an array of β-lactams, as well as vancomycin intermediate/resistant *S. aureus* (VISA and VRSA), resistant to one of last resort drugs, vancomycin. However, the spread of other antibiotic resistance determinants is not to be underestimated.

Continuing emergence of resistance determinants against novel drugs seems to be only a smaller part of a bigger picture. In fact, their rapid spread is what truly aggravates the problem in healthcare. When speaking about dissemination of resistance determinants, the role of mobile genetic elements (MGEs) and mechanisms determining their mobility should be considered. In staphylococci these include predominantly: transposons, insertion sequences, and integrons (Malachowa and Deleo, 2010; Domingues et al., 2012; Alibayov et al., 2014; Partridge et al., 2018), which shuffle genetic material within a cell and facilitate spread and acquisition of diverse determinants and virulence factors. For this sort of MGEs, is it necessary to be located in autonomously replicating units such as bacterial chromosome or other MGEs such as plasmids or phages. The latter are vectors for inter-cellular exchange of genetic material. Plasmids are usually incorporated by bacterial cells from the environment in an active manner by transformation (Fagerlund et al., 2014) or exchanged between cells either by conjugation (O’Brien et al., 2014; Ramsay et al., 2016) or phage transduction, which appears to be of particular relevance for plasmid transfer in staphylococci, including clinically relevant strains as USA300 (Varga et al., 2012, 2016). In turn, phages and likely phage-derived pathogenicity islands and staphylococcal cassette chromosomes seem to be mostly spread by transduction (Deghorain and Van Melderen, 2012; Scharn et al., 2013). Such a wide range of possibilities may justify a supposition that any resistance determinant may emerge anywhere and then be quickly spread among different bacterial species of diverse host or environment-specificity. Mass scale use of antibacterial drugs, especially in farming, increases the pressure for resistance determinants spread in natural environment and their exchange among free-living bacteria as well as animal commensals (Martinez, 2009; Fluit, 2012; He et al., 2014; Cuny et al., 2017; Kumar et al., 2017; Lau et al., 2017). Temporary colonization of humans by animal strains leading to the interaction with human microbiota or stable colonization (host jump) are not a rare phenomenon and the last part of the route (Armand-Lefevre et al., 2005; Voss et al., 2005; Bosch et al., 2016). Indeed, commensal strains of the same species or genus constitute a vast reservoir for antibiotic resistance determinants for those displaying pathogenic potential as it is observed for staphylococci (Tulinski et al., 2012; Hung et al., 2015; Morgenstern et al., 2016; Savini et al., 2016).

Apart from antibiotic resistance determinants, MGEs facilitate spread of heavy metal resistance and genes encoding virulence factors. The latter are a diverse group of mostly proteins and peptides that facilitate colonization and infection progress. These include for instance: cell-wall-anchored surface proteins interacting with host proteins, extracellular proteases, lipases, nucleases, super-antigens or cytotoxic proteins, and peptides (Marques et al., 1989; Takeuchi et al., 1999; Argudín et al., 2010; Berends et al., 2010; Bukowski et al., 2013; Thammavongsa et al., 2013; Wladyka et al., 2015; Bonar et al., 2016). Acquisition of genes coding for host-specific virulence factors, for instance in a plasmid, seems to be of importance for successful colonization of a new host by staphylococci and contributes to a phenomenon referred to as a host jump (Lowder et al., 2009; Fluit, 2012; McCarthy et al., 2014). Stable or transient livestock-to-human colonization events are continually being reported, particularly in farming environments where workers are exposed to frequent interaction with animals and the environment of their living. Importantly, such strains are also able to spread via human-to-human interaction (Armand-Lefevre et al., 2005; Voss et al., 2005; Huijsdens et al., 2006; Fluit, 2012; Wendlandt et al., 2013).

Nonetheless, emergence and dissemination of either antibiotic resistance determinants or virulence factors’ genes do not comprise the full picture. The last piece, out of the crucial ones, seems to be their stable maintenance. This is of particular importance to those encoded on low-copy-number plasmids (Sengupta and Austin, 2011; Dmowski and Jagura-Burdzy, 2013). Once acquired, in the absence of selective environmental pressure, plasmids carrying resistance determinants are either gradually lost or stably maintained when internal pressure is exerted. It has been conclusively demonstrated that such self-maintenance may be provided by toxin–antitoxin (TA) systems. In fact, the role of TA systems, in the context of stable genetic material maintenance in bacteria, is well-documented (Tsuchimoto et al., 1988; Sobecky et al., 1996; del Solar et al., 1998; Van Melderen and Saavedra De Bast, 2009; Bukowski et al., 2011, 2013). A number of recent reports point out their possible importance for drug resistance dissemination as well, for instance among enterococci (Moritz and Hergenrother, 2007; Rosvoll et al., 2010; Bukowski et al., 2017). However, it still seems that their role in this phenomenon has yet not received due attention.

In this study, complete sequences of 16 different plasmids, including 7 unknown to date, derived from 18 poultry *S. aureus* strains are reported along with a detailed analysis of distribution of antibiotic and heavy metal resistance determinants as well as other relevant factors among them. Moreover, the physiological effect of such determinants’ presence is carefully examined. Finally, a unique example is presented of plasmids’ shuffling and fragment exchange leading to accumulation of drug and heavy metal resistance determinants along with a TA operon in a likely mobilizable plasmid pPA3.

## Materials and Methods

### Bacterial Strains and Cultures

Poultry strains of *S. aureus* used in this study have been described previously elsewhere (Lowder et al., 2009; Polakowska et al., 2012; Bonar et al., 2016, 2018). The summary is included in Table 1. Bacteria were cultured in tryptic soy broth (TSB) or on tryptic soy agar (TSA) at 37°C unless otherwise described.

**Table 1:** *S. aureus* strains used in the study.

No.	Strain	Host	ST	Country	Year	Biosample accession
1.	ch3^1^	*Gallus gallus*	5	Belgium	1976	SAMN05853510
2.	ch5^1^	*Gallus gallus*	5	Belgium	1976	SAMN05853511
3.	ch8^1^	*Gallus gallus*	5	USA	1999	SAMN09846907
4.	ch9^1^	*Gallus gallus*	5	USA	1999	SAMN05853512
5.	ch10^1^	*Gallus gallus*	1342	USA	1999	SAMN09846908
6.	ch11^1^	*Gallus gallus*	692	UK	2006	SAMN09846909
7.	ch15^1^	*Gallus gallus*	385	Belgium	1976	SAMN09846910
8.	ch21^2^	*Gallus gallus*	5	Poland	2008	SAMN05853507
9.	ch22^2^	*Gallus gallus*	5	Poland	2008	SAMN05853508
10.	ch23^2^	*Gallus gallus*	5	Poland	2008	SAMN05853513
11.	ch24^2^	*Gallus gallus*	1	Poland	2008	SAMN05853514
12.	ch25^2^	*Gallus gallus*	5	Poland	2008	SAMN09846911
13.	pa2^1^	*Perdix perdix*	1346	UK	1997	SAMN09846912
14.	pa3^1^	*Perdix perdix*	692	UK	2016	SAMN05853515
15.	ph1^1^	*Phasianus colchicus*	1347	UK	Unknown	SAMN09846913
16.	ph2^1^	*Phasianus colchicus*	692	UK	Unknown	SAMN05853516
17.	tu1^1^	*Meleagris gallopavo*	5	UK	Unknown	SAMN09846914
18.	tu2^2^	*Meleagris gallopavo*	1	Poland	2008	SAMN09846915


*^*1*^Lowder et al. (2009). ^*2*^Polakowska et al. (2012). ST, sequence type according to multilocus sequence typing (MLST, Maiden et al., 1998).*

### Plasmid DNA Isolation

Three 2 ml samples of overnight cultures were collected and centrifuged at 5000 RCF. Supernatants were discarded and each pellet was re-suspended in 200 μl of EC buffer (Smith and Cantor, 1987) supplemented with 1 μl of RNAse A (10 mg/ml, Thermo Scientific) and 20 μl of lysostaphin (1 mg/ml, Madry et al., Unpublished), and then incubated in 37°C for 30 min. Samples were further subjected to plasmid isolation by alkaline lysis and purification on silica spin columns using Plasmid Mini kit (A&A Biotechnology) according to the manufacturer’s protocol. All lysates were pooled on one spin column and plasmid DNA was eluted in 50 μl of sterile, double-distilled, and filtered water. The obtained DNA concentrations ranged from 300 to 900 ng/μl.

### Sequencing and *de novo* Assembly

Plasmid sequencing was performed by Genomed (Warsaw, Poland) using the Illumina MiSeq system. The obtained data were processed in CLC Genomics Workbench, version 8.5.1 (Qiagen/CLC Bio). Reads were analyzed, trimmed, and filtered using Trim Sequences tool from the NGS Core Tools package, at default parameter values, and subsequently assembled into contigs using *de novo* Assembly tool. The assembly was carried out with scaffolding and automatic pair distance detection and default values for other parameters. Contig sequences were examined for homology to existing staphylococcal plasmids and their fragments using in-house Python 3 scripts, which utilized nucleotide BLAST from the NCBI BLAST+ toolkit, version 2.3.0 (Camacho et al., 2009), and ordered. In case of absence of an adequate reference sequence, the order of contigs was determined by PCR amplification. Each primer was designed to hybridize ca. 200 bp upstream each end of a contig. The primers were used in different combinations to assess the possible arrangement of contigs. The gaps were closed by Sanger sequencing.

### Sequence Comparative Analysis

Annotation was performed by using in-house Python 3 scripts and protein BLAST search on staphylococcal protein sequences available in NCBI Protein database, accessed on 14 January 2019, as well as RPS-BLAST search on NCBI Conserved Domain Database, accessed on 14 January 2019 (Marchler-Bauer et al., 2017). The annotation was manually revised and corrected. The complete and annotated plasmid sequences were deposited in GenBank. The accession numbers are included in Table 2. Plasmid pPA3 and homology of its fragments to existing plasmids were determined using nucleotide BLAST and visualized using Circos, version 0.69-3 (Krzywinski et al., 2009). Putative *oriT* mimics were searched using the core sequence of those from conjugative plasmids (O’Brien et al., 2015). The structure of *oriT* mimic was generated using mfold web server for single-stranded linear DNA at default parameters (Zuker, 2003) and visualized in CLC Main Workbench, version 12.0.1 (Qiagen/CLC Bio). Other visualizations were prepared using CLC Main Workbench and finished in GIMP image editor.

**Table 2:** Plasmids occurring among analyzed *S. aureus* strains.

No.	Plasmid	Strain	Length (bp)	Accession

	Group I			
1.	pAvX	ch5	17,258	MH785249
2.		ch8	17,257	MH785251
3.		ch9	17,259	MH785248
4.		ch10	17,257	MH785235
5.		ch21	17,257	CP017805
6.		ch22	17,257	CP017808
7.		ch23	17,259	MH785252
8.		ch25	17,257	MH785253
9.		pa2	17,257	MH785243
10.		ph2	17,220	MK388402
11.		tu1	17,257	MH785244
12.	pAvY	ch3	1,442	MH785238
13.		ch5	1,442	MH785239
14.		ch8	1,442	MH785240
15.		ch9	1,442	MH785241
16.		ch10	1,442	MK388404
17.		ch15	1,442	MH785259
18.		ch21	1,442	CP017806
19.		ch22	1,442	CP017809
20.		tu1	1,442	MH785234
21.	pAvY-B1	ch8	1,435	MH785257
22.		ch23	1,435	MH785242
23.		ch25	1,435	MH785237
24.		pa2	1,435	MK388403
25.	pAvY-B2	ch23	1,475	MH785231
26.		ch25	1,475	MH785225

	**Group II**			

27.	pLUH02	ch3	27,269	MH785250
28.		ch5	27,261	MH785232
29.	pMW2	ch24	20,653	MH785254
30.		tu2	20,630	MH785247
31.	pRIVM1295-2	ph1	2,992	MH785226
32.	pRIVM4390	ch24	4,397	MH785228
33.		tu2	4,397	MH785256
34.	pSAP060B	ch8	4,440	MH785230
35.		ph1	4,498	MH785224
36.		tu1	4,440	MH785255

	**Group III**			

37.	pCH8	ch8	2,036	MH785245
38.	pCH11	ch11	3,259	MH785227
39.	pPA3	pa3	26,968	MH785229
40.	pPH1-1	ph1	30,962	MH785236
41.	pPH1-2	ph1	17,747	MH785258
42.	pPH1-3	ph1	3,605	MH785246
43.	pPH2	ph2	16,747	MH785233


*The top part of the table lists pAvX and pAvY plasmids specific for poultry strains (group I), including two newly uncovered pAvY homologs, namely pAvYB1 and pAvY-B2; the middle, plasmids previously recorded in S. aureus strains of different origin (group II); the bottom, entirely novel plasmids reported in this study (group III).*

### Phylogenetic Analyses

Multiple sequence alignments were prepared using CLC Main Workbench with the following parameter values for both DNA and protein sequences: gap open cost, 20.0; gap extension cost, 1.0; end gap cost, free; alignment mode, very accurate. Phylogenetic trees were constructed and visualized in CLC Main Workbench for gapless segments of multiple sequence alignments using maximum-likelihood phylogeny tool with the following parameter values: construction method, neighbor joining; nucleotide substitution model, general time reversible, GTR; transition/transversion ratio, 2.0; include rate variation, yes; number of substitution rate categories, 8; gamma distribution parameter, 1.0; estimate substitution rate parameter(s), yes; estimate topology, yes; estimate gamma distribution parameter, yes; perform bootstrap analysis, yes; and replicates, 100.

### Detection of Alternative pAvY Plasmids

The detection of pAvY, pAvY-B1, and pAvY-B2 plasmids was carried out by PCR reaction using primers listed in Table 3. RUN DNA Polymerase (A&A Biotechnology) was used according to the manufacturer protocol with elongation time 1 min and annealing temperature 60°C.

**Table 3:** Primers used for detection of pAvY, pAvY-B1, and pAvY-B2 plasmids.

Name	Sequence	Length (bp)	GC content (%)	*T*_M_ (°C)
pAvY-F	5′-GCAATTATTCTGAAGTAGCTG-3′	21	38	55.4
pAvY-B1-F	5′-GGTAATTATTCTGATTTGAGTAG-3′	23	30	55.5
pAvY-B2-F	5′-GTACTTTTGAAGAGCTTAAATAC-3′	23	30	55.5
pAvY-R	5′-CATAAACAATCAACACAAAGAG-3′	22	32	54.7
pAvY-B1-R	5′-AATGTCATCCTAATTTCATTCTC-3′	23	30	55.5
pAvY-B2-R	5′-CTCGGCATAAATGAGAATGC-3′	20	45	56.4


**T*_*M*_, melting temperature adjusted to 50 mM salt concentration (Kibbe, 2007).*

### Antibiotic Resistance Determination

A few bacterial colonies were sampled from an overnight agar plate, suspended in 0.9% NaCl solution and diluted to 0.5 density in McFarland scale using DEN-1B McFarland Densitometer (Biosan). Suspensions were streaked on Mueller Hinton Agar (MHA) plates (Oxoid) using Eddy Jet 2 automatic spiral plater (IUL) in the Lawn 3000 mode. Plates were dried shortly and MIC Test Strips (Liofilchem) were carefully placed on them. The incubation conditions and measurement were carried out according to the manufacturer’s protocol and the interpretation of the results according to EUCAST 2015 (European Committee for Antibiotic Susceptibility Testing) and CLSI 2015 (Clinical and Laboratory Standards Institute) recommendations, which in most cases meant that the results were interpreted after 24 h incubation in 37°C. The analyzed strains always fell into the sensitive or resistant group regardless of whether CLSI or EUCAST interpretation was followed. For the lack of CLSI or EUCAST interpretation for streptomycin, a previous report was used to interpret the results (Archer, 1978).

### Heavy Metal Resistance Determination

Fresh liquid cultures were prepared by dilution of overnight ones at 1:100 ratio followed by incubation in the same conditions to reach the optical density at 600 nm close to 0.6. All cultures were adjusted to the optical density of 0.6 and diluted 100-fold. 100 μl samples were transferred to 96-well plates and mixed with 100 μl of heavy metal inorganic compound (CdCl_2_, Na_2_HAsO_4_) solutions in TSB. Twofold serial dilutions in the concentration range from 10 to 160 μM in case of CdCl_2_ and 10-fold dilutions in range of 20 μM to 20 mM in case of Na_2_HAsO_4_ were used. Subsequently plates were incubated at 37°C for 20 h and the minimal inhibitory concentration (MIC) was determined as the lowest heavy metal compound concentration where no bacterial growth was observed. *S. aureus* RN4220 was used as a reference non-resistant to heavy metals. The results were interpreted according to previous reports (Ji and Silver, 1992; Crupper et al., 1999; Chudobova et al., 2015).

## Results

Among 18 strains that were analyzed in the present study 43 occurrences of 16 different plasmids have been reported. Based on their novelty and prevalence among analyzed strains, the plasmids were divided into three distinct groups: (I) poultry-associated plasmids; (II) plasmids of previously known sequences occurring in *S. aureus* strains of diverse host-specificity; and (III) plasmids entirely unknown to date and characterized in this study (Table 4).

**Table 4:** An overview of plasmid occurrence among 18 poultry strains of *S. aureus*.

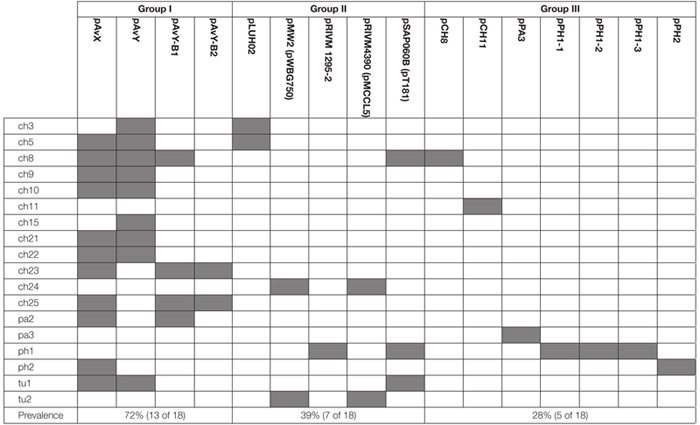

*The first group of columns contains pAvX and pAvY plasmids specific for poultry strains (group I); the middle, plasmids previously recorded in *S. aureus* strains of different origin (group II); the last, newly uncovered plasmids reported in this study (group III). Presence of plasmids is marked by gray fill. Prevalence of plasmids belonging to a particular group among analyzed strains is given at the bottom.*

### Poultry-Associated pAvX and pAvY (Group I)

**FIGURE 1:**
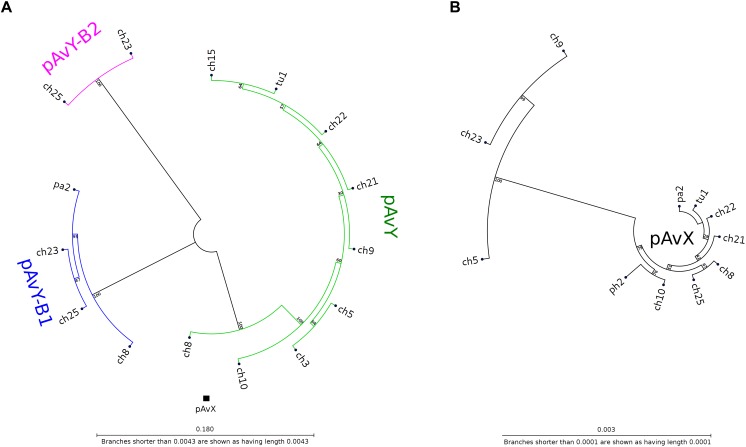
Phylogenetic trees of pAvY group and pAvX plasmids. Panel **(A)**: based on the phylogenetic analysis, pAvY group plasmids might be divided into three distinctive subgroups: common pAvY next to less frequently occurring pAvY-B1 and pAvY-B2. Panel **(B)**: a corresponding tree for pAvX plasmids. These plasmids are highly conserved among all strains when compared to pAvY group. Note that for readability the tree is presented in 60-fold bigger scale. A small black box under the tree in the panel **A** shows its size in the scale used for pAvY plasmids. On each branch the bootstrap value is given.

**FIGURE 2:**
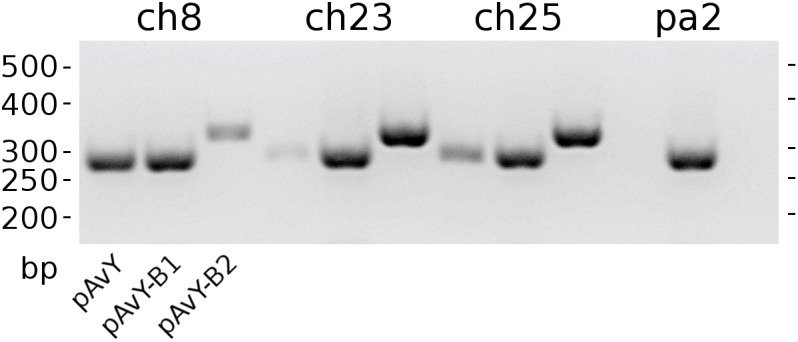
The occurrence of pAvY group plasmids. Expected products’ lengths are: 278 bp for pAvY and pAvY-B1; 321 bp for pAvY-B2. Specific PCR products are visible for pAvY and pAvY-B1 for ch8 strain; pAvY-B1 and pAvY-B2 for ch23 and ch25 strains; and pAvY-B1 for pa2 strain. For ch8, ch23, and ch25, there are clearly weaker signals from PCR products for the remaining variants, which likely are unspecific products.

The first group contains known plasmids characteristic for poultry strains. These are pAvX and pAvY (Lowder et al., 2009) as well as two of pAvY’s variants (pAvY-B1 and pAvY-B2), uncovered in this study, which clearly belongs to distinct phylogenetic groups (Figure 1). Remarkably, all three pAvY group variants occur independently and can co-exist next to each other in the same strain, which was independently verified by PCR (Figure 2) to exclude unlikely, however possible, NGS assembly artifacts. In general, the poultry-associated group contains the most frequently occurring plasmids with total prevalence of 72% of the analyzed strains, 61% for pAvX, and 67% for pAvYs. Although frequently occurring, these plasmids do not carry any resistance determinants. However, their presence correlates with virulence (Lowder et al., 2009; Polakowska et al., 2012).

As regards virulence factors, in pAvX there are genes coding for those of well-documented role (Table 5) such as staphopain A2 (Wladyka et al., 2011a,b; Kalińska et al., 2012) and lysophospholipase (Doery et al., 1965; Marques et al., 1989; Daugherty and Low, 1993). Next to them there is also an operon of *pemIK-Sa1*, which is the most frequently occurring in staphylococcal plasmids TA system of *mazEF*/*pemIK* family (Bukowski et al., 2017) and belongs to class II TA systems with the toxin being a sequence-specific RNase (Bukowski et al., 2013).

**Table 5:** Distribution of antibiotic and heavy metal resistance determinants, genes of virulence factors, as well as genetic elements relevant for their maintenance among the three groups of analyzed plasmids.

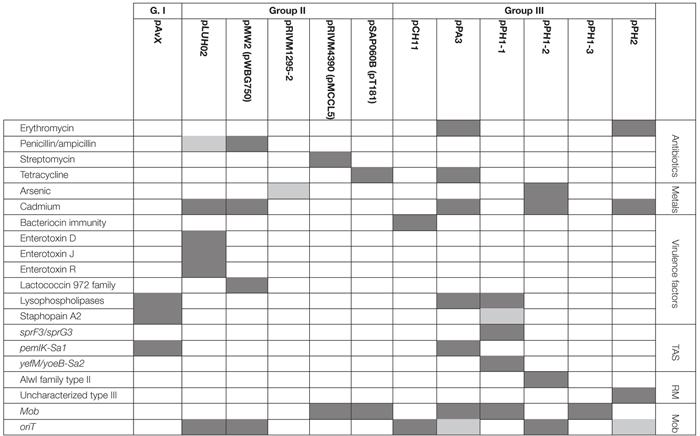

*Presence of genetic elements is marked by dark-gray fill. Light-gray fill denotes unique cases: pLUH02 provides resistance only in ch5 strain, in ch3 the open-reading frame of β-lactamase is truncated; for pRIVM1295-2 the activity of a putative arsenite methyltransferase could not be distinguished from the activity of the canonical resistance system in pPH1-2; in pPH1-1, there is only a partial open-reading frame of *scpA2*. In case of *oriT*, light-gray denotes the presence of its mimic. TAS, toxin–antitoxin system; RM, restriction–modification system; Mob, mobility-related elements.*

### Plasmids of Known Sequences (Group II)

The second group includes five known plasmids that have been reported so far in *S. aureus* strains of different origin. The prevalence of these plasmids reaches 39% of analyzed strains.

While the prevalence rate of 39% for this group is by more than one-third lower than that for pAvX and pAvYs (72%), these plasmids are frequent carriers of antibiotic resistance determinants against drugs from groups of aminoglycosides (streptomycin, pRIVM4390), β-lactams (penicillin, pMW2), macrolides (erythromycin, pRIVM1295-2), and tetracyclines (tetracycline, pSAP060B), next to resistance determinants to cadmium (pLUH02 and pMW2) or putatively to arsenic (pRIVM1295-2). Except the strain ch3, for the reasons discussed further, all the remaining carrier strains displayed corresponding resistant phenotypes (Table 5 and Supplementary Tables S1, S2).

Regarding genes of known virulence factors, only pLUH02 carries a cluster of genes of enterotoxins D, J, and R. In pMW2, however, there is a gene of a yet uncharacterized lactococcin 972 family bacteriocin. Bacteriocins facilitate the producer host or environment colonization by eliminating other bacteria. Nevertheless, some bacteriocins exhibit properties of virulence factors (Wladyka et al., 2015). The neighborhood of the aforementioned gene suggests that it is a part of the whole operon, providing immunity proteins and transporters necessary for excretion. Interestingly, plasmids pLUH02 and pMW2 possess *oriT*, which potentially renders them mobilizable.

### Plasmids Unknown to Date (Group III)

**FIGURE 3:**
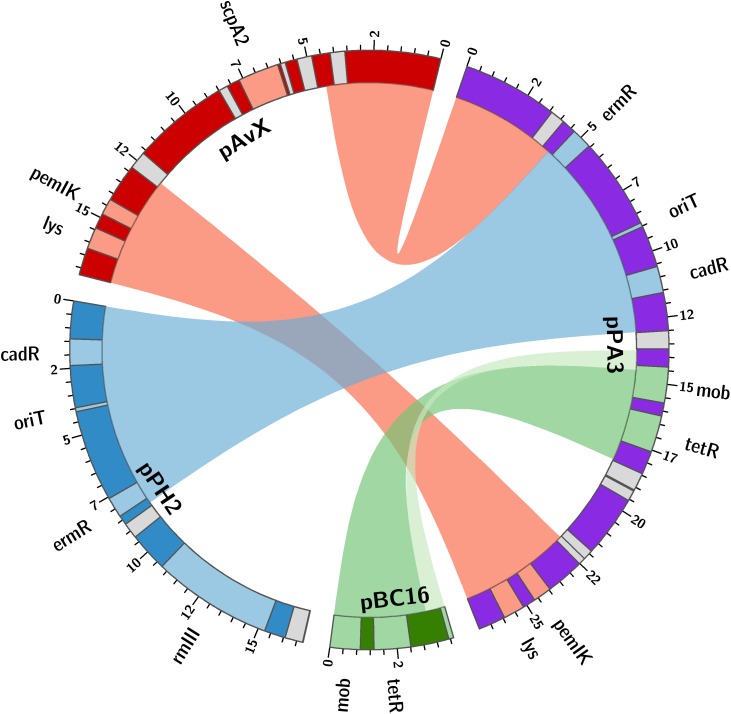
The structure of pPA3 plasmid. The plasmid is composed of elements originating from three different plasmids: common for staphylococcal poultry strains pAvX donates lysophospholipase gene (*lys*) and *pemIK-Sa1* TA system operon (*pemIK*); pPH2, reported in this study, donates a functional erythromycin and cadmium resistance determinants (*ermR* and *cadR*) as well as the uncovered in this study *oriT* mimic (*oriT*); pBC16 is entirely incorporated into pPA3 and next to mobilization protein gene (*mob*) provides a functional tetracycline resistance determinant (*tetR*). The uncharacterized type III RM system (*rmIII*) and staphopain A2 (*scpA2*) operons are also depicted in pPH2 and pAvX, respectively. Light gray boxes denote genes of transposases and recombinases.

The last group consists of plasmids that have not as yet been reported. Prevalence of these plasmids among the analyzed strains reaching 28% is the lowest when compared to the previous groups. However, these are as frequent carriers of genes and determinants, whose prevalence is analyzed in this study, as the second group. Their sequences have been entirely unknown to date with the exception of pPA3. The sequence of pPA3 is, to a considerable extent, a mosaic of sequences derived from other plasmids, each of them provides a unique element and each is flanked by shuffle-inducing sequences such as genes of transposases or invertases (Figure 3).

As regards resistance determinants, pPA3 carries two of such, against erythromycin and tetracycline. The erythromycin resistance determinant is located in a fragment originating from another unknown to date and reported in this study plasmid, namely pPH2, whereas the tetracycline resistance determinant originates from pBC16, a broad-host plasmid. Both, pPA3 and pPH2, contain determinants of cadmium resistance. Cadmium resistance determinants, together with one of resistance against arsenic, are also located in pPH1-2.

Concerning genes of possible virulence factors, pCH11 carries an immunity determinant against a bacteriocin. However, the authors were unable to find any gene coding for a bacteriocin in this plasmid, which would mean the existing gene provides immunity to one or more bacteriocins produced by other strains. Apart from this gene, pCH11, pPA3, and pPH1-1 possess one encoding lysophospholipase of very high similarity to the one encoded in pAvX.

As for TA systems and functionally related to them restriction–modification (RM) systems (Makarova et al., 2013; Mruk and Kobayashi, 2014), pPA3 contains an operon of *pemIK-Sa1* localized in a fragment originating from pAvX. Strikingly, pPH1-1 possesses operons of two unrelated TA system. These are *yefM/yoeB-Sa2*, which is also class II TA system with the toxin being an RNase (Yoshizumi et al., 2009; Nolle et al., 2013), and a homolog of *sprF3*/*sprG3*, which is a class I TA system with the toxin being a bactericidal peptide and the antitoxin an antisense RNA for the toxin transcript (Germain-Amiot et al., 2018; Riffaud et al., 2018). Noteworthy, whereas *pemIK-Sa1* prevail mostly in plasmids (Bukowski et al., 2017), *yefM/yoeB-Sa2* is mostly chromosome-located (Yoshizumi et al., 2009; Nolle et al., 2013) and *sprF/sprG* type systems have been reported so far in staphylococcal pathogenicity islands that carry virulence factors and antibiotic resistance (Pichon and Felden, 2005; Riffaud et al., 2018). Plasmids pPH1-2 and pPH2 carry operons of RM systems. The former of type II and AlwI family, whose close homologs are present in a few known staphylococcal genomes, and more distant ones in genomes of other closely-related Gram-positive genera of *Firmicutes* phylum such as *Bacillus*, *Enterococcus*, and *Streptococcus*. The latter a type III RM system whose closest homologs may be found in *Salinicoccus*, a genus of free-living, halotolerant bacteria from *Staphylococcaceae* family, and more distant ones in genera of *Bacillus*, *Enterococcus*, *Geobacillus*, *Staphylococcus*, and *Streptococcus*. Neither of these RM systems nor their close homologs have yet been characterized.

Regarding the possibility of horizontal transfer, pCH11, pPA3, pPH1-2, and pPH2 are likely mobilizable as all possess *oriT* or its mimic. Plasmid pPA3 also contains a gene of mobilization protein. Notably, in case of pPA3, *oriT* mimic is located within a fragment originating from pPH2 but the gene of mobilization protein comes from pBC16 (Figure 3). Two other plasmids co-existing with pPH1-2, which are pPH1-1 and pPH1-3, encode mobilization protein *in trans* in respect to pPH1-2.

## Discussion

Plasmids seem to be the principle means of dissemination of antibiotic and heavy metal resistance as well as host-specific virulence factors. However, the contemporary abundance of genomic data does not necessarily facilitate tracing their spread in bacterial populations. The main reason is the incompleteness of the genomic sequences. Out of 10,268 assemblies available for *S. aureus* on 29 January 2019, only 6.3%, are complete sequences (Supplementary Data S1). The remaining genomic sequences are deposited as sets of contigs assembled based on NGS raw data. For contigs it is challenging or even impossible to assess whether a genome contains plasmids. As a result, for more than 10,000 genomic sequences there are only 430 complete plasmid sequences available for *S. aureus* today (Supplementary Data S1). The magnitude of this estimation closely corresponds with recent reports (O’Brien et al., 2015; Kwong et al., 2017) and has not changed for last 7 years (Lozano et al., 2012).

The frequent presence of group I plasmids, namely pAvX and pAvY, in poultry strains has been reported before (Lowder et al., 2009). The former is a carrier of an operon encoding for cysteine protease staphopain A2 (Takeuchi et al., 1999, 2002; Wladyka et al., 2011a; Kalińska et al., 2012). This protease has been suggested to be a host-specific virulence factor for poultry strains and the transfer of its operon in pAvX to strains of human origin to facilitate a documented interspecies jump from a human to a poultry host (Lowder et al., 2009). The presence of TA system *pemIK-Sa1* likely stabilizes its maintenance as the system has been demonstrated to display required properties (Bukowski et al., 2013). Conversely, homologs of group II plasmids, pRIVM4390 (pMCCL5) and pRIVM1295-2 (GenBank accessions CP013623 and CP013618), were reported recently in an opposite context, namely in livestock-associated MRSA (LA-MRSA) strains transmitted from farmed animals to humans in Netherlands, which dated back to 2003 (Bosch et al., 2016). It is noteworthy that pRIVM1295-2 found here in ph1 strain, which belongs to sequence type 1347 (ST1347) according to multilocus sequence typing (MLST, Maiden et al., 1998), was reported before by Lowder et al. (2009) regarding a single human-to-poultry host jump and radiation among broiler chicken of unique to Poland, human ST5 clonal lineage that was roughly estimated to have occurred around 1970s. Two other strains analyzed in our study, ch24 and tu2 (ST1), are MRSA isolated in Poland in 2008 (Polakowska et al., 2012) and carry pRIVM4390. It suggests that these two plasmids are spread among LA-MSSA as well as LA-MRSA and have migrated among different lineages for many decades contributing to resistance determinants dissemination. Plasmid pRIVM4390 was linked by Bosch et al. (2016) to resistance against aminoglycosides: neomycin and kanamycin; and in this study against streptomycin as well (Table 5). The other plasmid, pRIVM1295-2, was not documented to provide any antibiotic resistance. However, we found that one of its genes codes for a protein distantly similar to arsenite methyltransferases, which may comprise an accessory detoxification system to the one present in group III plasmid pPH1-2 of the same strain ph1 (Table 5), which provides resistance against arsenite as well as arsenate. Strain ph1 indeed displays elevated resistance to arsenate (Supplementary Table S2).

The analyzed in this study group II encompasses plasmids of known sequences that were originally reported in human isolates. The plasmid named here pSAP060B carries a tetracycline resistance determinant and occurs in three strains: ch8 (ST5), ph1 (ST1347), and tu1 (ST1). Its exact copy (GenBank accession GQ900417) was reported originally in 2010 in *S. aureus* SAP060B strain in a research focused on emergence of plasmid-related resistance against non-β-lactams among isolates of *S. aureus* USA300 strain (McDougal et al., 2010). *S. aureus* USA300 (ST8) remains of a particular interest as it is a community-associated MRSA (CA-MRSA) responsible for invasive infections across the United States (Moran et al., 2006; Tenover et al., 2006). The sequence of pSAP060B is nearly identical to one of the earliest sequenced staphylococcal plasmid pT181, GenBank accession J01764 (Khan and Novick, 1983). In both cases, it was reported for human clinical subjects and appears to be a frequently occurring plasmid among *S. aureus* clinical isolates (Águila-Arcos et al., 2017). Nevertheless, pT181 has been reported in human-origin poultry strains before (Lowder et al., 2009), and in this study among three strains of different sequence types, which demonstrates its frequent prevalence among unrelated lineages. A similar example is plasmid pMW2 (pWBG750) carrying a penicillinase gene. It was first reported in a study on the whole genome sequence of highly virulent human CA-MRSA strain MW2 (ST1), which was a cause of fatal septicemia and septic arthritis in a 16-month-old child in the United States in 1998 (Baba et al., 2002). Here we found the plasmid in two aforementioned LA-MRSA strains, namely ch24 and tu2, which markedly are of the same sequence type (ST1). This shows that plasmids that provide resistance determinants and were initially associated with human strains may be stably maintained in the bacterial population after a host jump, spread among livestock, and contribute to this vast reservoir of resistance determinants (Armand-Lefevre et al., 2005; Voss et al., 2005; He et al., 2014; Lau et al., 2017). The next plasmid that supports this conclusion is pLUH02, which we found in ch3 and ch5 strains of the ST5 lineage originating from a human-to-poultry host jump (Lowder et al., 2009). A homolog of pLUH02 (GenBank accession FR714929) has been uncovered among multi-resistant methicillin-susceptible *S. aureus* (MR-MSSA) isolates from a clinical outbreak in Sweden in 2009 (Lindqvist et al., 2009, 2012). Despite the fact that the isolates were MSSA, the authors argue that they likely originated from MRSA in which the major extent of SCCmec cassette, a MGE carrying determinants of resistance against multiple β-lactams, was lost from the chromosome. For ch5, pLUH02 provides penicillin/ampicillin resistance. In case of ch3 this resistance is not manifested, which is likely a result of a single nucleotide deletion within β-lactamase gene. Nevertheless, in both cases, pLUH02 possesses functional determinants providing resistance against cadmium and an array of genes coding for virulence factors, namely for enterotoxins D, J, and R (Casman et al., 1967; Zhang et al., 1998; Omoe et al., 2003). A series of plasmids carrying the same cluster of enterotoxin genes was reported before Lindqvist et al. (2012) by Omoe et al. (2003) as pIB485-like plasmids. Indeed, pLUH02 belongs to this group (Shearer et al., 2011). Worth mentioning in this context is also a novel plasmid pCH8. It is the most similar to a small plasmid pHSSA0406 (GenBank accession KR870311) described recently in a study analyzing distribution of fosfomycin resistance among MRSA strains isolated in a clinical setting from blood or cerebrospinal fluid (Fu et al., 2016). Nevertheless, the determinant of fosfomycin resistance is lost from pCH8.

Next to known plasmids, previously unknown ones, comprising the group III, are reported in this study as a considerable part of the whole plasmid pool, which may suggests that there is still a significant number of yet uncovered plasmids in *S. aureus* strains. Among 16 different plasmid sequences reported here, 7 are novel. Among these sequences, there are a few of particular interest. The first one is the newly established group of pAvY plasmids, which replaces what was reported before as one pAvY plasmid (Lowder et al., 2009). The meaning of plasmids from pAvY group for *S. aureus* strains is elusive. The originally reported pAvY carries only two genes. One coding for a short hypothetical protein, unique to pAvY, and another for a replication protein. Two remaining variants of the plasmid uncovered in this study, namely pAvY-B1 and B2, carry only the gene for the replication protein. Sequence relatedness of these plasmids is strictly reflected in sequences of this protein (Supplementary Figure S1), which may render their replication machineries independent and could explain their stable co-maintenance. The role of pAvY group plasmids remains a mystery. They appear not to carry any relevant genes, yet frequently occur in poultry strains. Presumably they support the maintenance of pAvX plasmid they co-exist with. It has been observed in this study that 10 out of 11 strain carrying pAvX carry one or two plasmids from pAvY group as well (Table 4), which remains consistent with the original study (Lowder et al., 2009). Nevertheless, a possibility of pAvYs being simply selfish genetic elements is also feasible.

Regarding the mechanism of antibiotic resistance and other determinants dissemination, strikingly, a considerable number of plasmids reported in this study, i.e., 6 out of 16, that belong to the group II and III, carry an *oriT* element or its mimic. Such a sequence may render plasmids mobilizable in the presence of a conjugative plasmid, which facilitate their transfer and accelerate their spread among bacterial cells. In many reported cases a gene of mobilization protein (*mob*), also known as nicking relaxase, is also present. The gene is provided either *in cis*, as the *oriT* mimic in pPA3, or *in trans* in co-existing plasmids, as in the pair of pMW2/pRIVM4390 or among pSAP060B/pPH1-1/pPH1-3 and pPH1-2 plasmids. However, alongside *oriT* and a mobilization protein the presence of a conjugative plasmid carrying accessory genes, as one for a coupling protein, and an essential, large gene array encoding a type IV secretion system (T4SS) responsible for creating the mating pore and the physical transfer, is also required (O’Brien et al., 2015; Ramsay and Firth, 2017). Nevertheless, it is clear that a considerable number of plasmids reported in this study are potentially mobilizable. This shows why the spread of antibiotic resistance and other determinants may progress quickly as it is observed (O’Brien et al., 2014; Ramsay et al., 2016; Ramsay and Firth, 2017).

**FIGURE 4:**
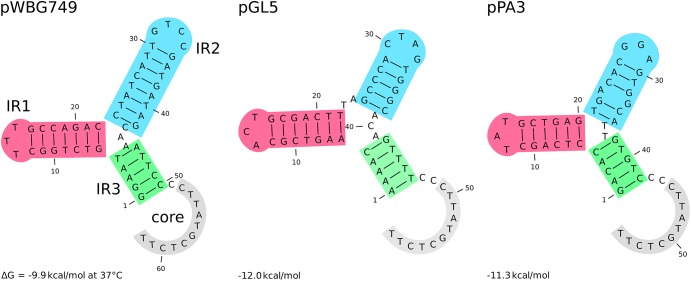
The secondary structure of different *oriT* elements. From the left: *oriT* and its mimic derived, respectively, from the conjugative plasmids pWBG747 and mobilizable plasmid pGL5 followed by the novel *oriT* mimic found in the fragment shared by the uncovered in this study pPH2 and pPA3 plasmids. Three inverted repeats and the core part are highlighted. The secondary structure and their Gibbs free-energy are similar across presented elements and others reported before (O’Brien et al., 2015).

Among group III plasmids, which are the newly uncovered in this study, pPA3 is exceptionally compelling. In-depth analysis of its sequence demonstrates the major mechanisms that underpin resistance determinants and virulence factors dissemination brought up in this work. The fragment of pAvX, the plasmid of group I common among poultry strains, provides the gene of lysophospholipase and the operon of *pemIK-Sa1* TA system. Another uncovered in this study group III plasmid pPH2 donates erythromycin and cadmium resistance determinants as well as an *oriT* mimic, whereas pBC16 is a source of a determinant of tetracycline resistance and a gene of mobilization protein. Strikingly, pBC16, which does not occur autonomously in the analyzed strains, is a broad-host plasmid that has been recorded so far in genera of *Bacillus*, *Enterococcus*, *Staphylococcus*, and *Streptococcus*. Hence, the observed sequence of pPA3 is a prime example of resistance determinants accumulation by interspecies transfer and DNA shuffling. Markedly, pAvX-derived *pemIK-Sa1* TA system likely stabilizes the maintenance of pPA3 together with the resistance determinants it carries and may contribute to their dissemination in bacterial populations, similarly as *yefM/yoeB-Sa2* and *sprF3/sprG3* in pPH-1. Such a process might be further accelerated by the presence of *mob* from pBC16 and the *oriT* mimic from pPH2, which may render pPA3 mobilizable. Mimics of *oriT* are sequences that apart from the core fragment differ from the original *oriT* sequences derived from conjugative plasmids. However, in a form of single-stranded DNA they assume a structure mimicking the structure of original *oriT* sequences. Hence, it is suggested that their presence renders a far greater number of plasmids mobilizable than had initially been expected based on the prevalence of *oriT* sequences from conjugative plasmids (O’Brien et al., 2015). Here a completely novel sequence of *oriT* mimic is reported in the shared fragment of unknown to date staphylococcal pPH2 and pPA3 plasmids (Figure 4).

## Conclusion

The role of plasmids in dissemination of antibiotic and heavy metal resistance as well as host-specific virulence factors is commonly recognized. Unfortunately, a vast number of sequencing projects do not aim for complete sequence determination, which frequently requires a considerable amount of extra workload. In consequence, the number of known complete plasmid sequences is disproportionately low. To address this problem, we undertook an effort to determine complete plasmids sequences from 18 *S. aureus* strains of poultry origin. The results show that most of plasmids occurring in the analyzed strains and reported beforehand are being found in clinical isolates of strains typical for humans or human ones of livestock origin. This shows that livestock-associated staphylococci are a significant reservoir of resistance determinants and virulence factors which are spread to human strains. Strikingly, nearly half of the plasmids presented here were unknown to date. In this group we found a plasmid named here as pPA3, which is a unique example of accumulation of resistance determinants and virulence factors likely stabilized by the presence of a TA system. These facts clearly demonstrate that there is a pressing need for studies aimed at complete plasmid sequence determination.

## Data Availability

The datasets generated for this study can be found in GenBank.

## Auhtor Contributions

MB and BW designed the study. MH grew bacterial cultures and isolated plasmid DNA. RP and MB analyzed the NGS data, performed *de novo* assembly, and determined the final sequence of pPA3 plasmid. MB and RZ-P performed *de novo* assembly and determined the final sequences of the remaining plasmids. MB prepared annotations and carried out comparative sequence analyses. AM did antibiotic and heavy metal resistance screening. All authors analyzed and interpreted the results. MB prepared figures and tables. MB and BW wrote the manuscript. All authors revised the manuscript and agreed to be accountable for all aspects of the presented work.

## Conflict of Interest Statement

The authors declare that the research was conducted in the absence of any commercial or financial relationships that could be construed as a potential conflict of interest.
