# Supplementary material for: Prevalence of Antibiotic and Heavy Metal Resistance Determinants and Virulence-Related Genetic Elements in Plasmids of Staphylococcus aureus
Source: Front Microbiol. 2019 Apr 24;10:805. doi: 10.3389/fmicb.2019.00805 (PMC6491766; doi:10.3389/fmicb.2019.00805)
Supplement: Supplementary file 1 [file Data_Sheet_1.pdf]

Prevalence of antibiotic and heavy metal resistance determinants and virulence-related genetic elements in plasmids of *Staphylococcus aureus*

Supplementary materials

Michal Bukowski[1#], Rafal Piwowarczyk[1], Anna Madry[1], Rafal Zagorski-Przybylo[1], Marcin Hydzik[1], Benedykt Wladyka[1#]

[1] Department of Analytical Biochemistry, Biophysics and Biotechnology, Jagiellonian University, Krakow, Poland

# corresponding authors:

Benedykt Wladyka
benedykt.wladyka@uj.edu.pl

Michal Bukowski
m.bukowski@uj.edu.pl

Contents

Supplementary data 1………….……………………………… 2
Supplementary table 1………………………………………. 3
Supplementary table 2………………………………………. 4
Supplementary figure 1……………………………………… 5
References…………………………………………………… 6

Suppl. Data 1. Determination of the number of *S. aureus* known complete plasmids. Sequences of complete plasmids of *S. aureus* were searched for in NCBI Nucleotide database, accessed on 2019-01-29, using the following query: "*Staphylococcus aureus" [Organism] AND "plasmid" [Title] AND "complete" [Title]*. GenBank sequences of 987 found entries were retrieved. 889 sequences marked as circular DNA sequences were used in the downstream analysis. These sequences were clustered with CD-HIT (Fu et al., 2012; Li and Godzik, 2006) at 90% identity threshold and the requirement of 100% query-to-subject and subject-to-query coverage, which allowed to determine the number of known complete plasmid sequences derived from *S. aureus* as 430. The number of available *S. aureus* genome assemblies and their status was directly inspected in NCBI GenBank database FTP site on 2019-01-29.

Suppl. tab. 1. Antibiotic resistance screening of the analysed strains. Minimal inhibitory concentrations (MIC) determined for strains with plasmids carrying respective determinants. In ch3 the presence of pLUH02 does not entail resistance to either ampicillin or penicillin since the open reading frame of β-lactamase is N-terminally truncated. The remaining values indicate resistant phenotypes.

Strain	MIC [µg/ml]				
	Ampicillin	Erythromycin	Penicillin	Streptomycin	Tetracycline
ch3	0.19	-	0.09	-	-
ch5	1.50	-	0.75	-	-
ch8	-	-	-	-	32.00
ch24	16.00	-	24.00	384.00	-
pa3	-	> 256.00	-	-	32.00
ph1	-	-	-	-	16.00
ph2	-	> 256.00	-	-	24.00
tu1	-	-	-	-	48.00
tu2	16.00	-	32.00	256.00	-

Suppl. tab. 2. Heavy metal resistance screening for the analysed strains. Minimal inhibitory concentrations (MIC) determined for strains with plasmids carrying respective determinants. Each test was carried out in two replicates. *S. aureus* RN4220 was used as a sensitive to heavy metals control.

Strain	MIC			
	Cd^{2+} [μM]		As^{5+} [mM]	
	I	II	I	II
ch3	40	80	-	-
ch5	40	80	-	-
ch24	80	80	-	-
pa3	80	80	-	-
ph1	40	80	-	-
ph2	80	80	> 10	> 10
RN4220	10	10	1	1

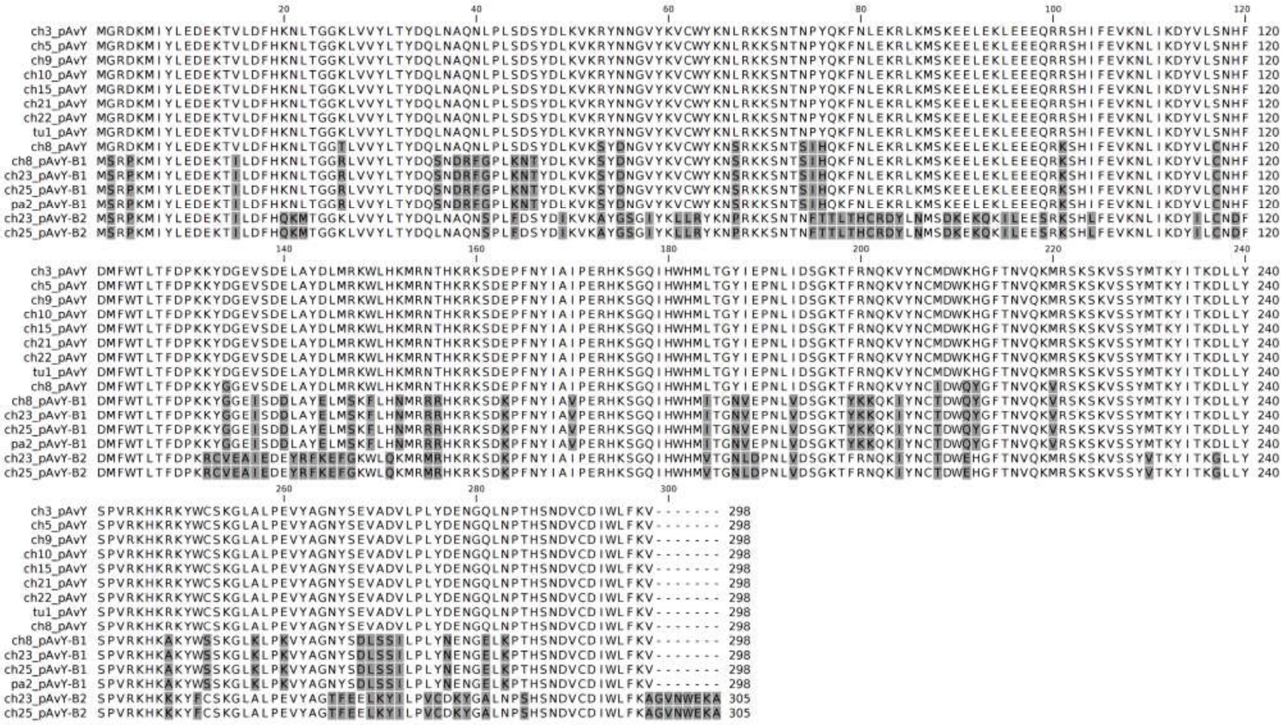

Suppl. fig. 1. The alignment of the replication protein of all pAvY group plasmids. The overall protein sequence is conserved, however there are variable regions whose sequences correlate with plasmid affiliation to pAvY, pAvY-B1 or pAvY-B2 group. The differentiating residues are highlighted.

References

Fu, L., Niu, B., Zhu, Z., Wu, S., and Li, W. (2012). CD-HIT: accelerated for clustering the next-generation sequencing data. *Bioinformatics* 28, 3150–3152. doi:10.1093/bioinformatics/bts565.

Li, W., and Godzik, A. (2006). CD-HIT: a fast program for clustering and comparing large sets of protein or nucleotide sequences. *Bioinformatics* 22, 1658–1659. doi:10.1093/bioinformatics/btl158.
